# Effect of Germination on the Nutrient Profile, Antioxidant Components, Antinutritional Constituents, and Functional Characteristics of Barnyard Millet ( *Echinochloa frumentacea* )

**DOI:** 10.1002/fsn3.71618

**Published:** 2026-03-17

**Authors:** Aina Chaudhary, Meena Kumari, Rachna Sehrawat, Lokesh Kumar

**Affiliations:** ^1^ Amity Institute of Food Technology AMITY University Noida Uttar Pradesh India; ^2^ Department of Food Process Engineering National Institute of Technology Rourkela Rourkela Odisha India; ^3^ Department of Wine Food and Molecular Biosciences, Lincoln University Lincoln New Zealand

**Keywords:** antinutritional factor, bioactive compounds, functional properties, germination, millets

## Abstract

Barnyard millet ( *Echinochloa frumentacea* ) is a nutrient‐dense, gluten‐free grain with recognized health benefits; however, its utilization is constrained by the presence of phytate, tannins, and trypsin inhibitor activity. This study examined the influence of germination (24–72 h) on the nutritional composition, antinutritional constituents, bioactive compounds, and functional properties of barnyard millet. Germination significantly altered the nutrient composition, increasing protein content (9.21–13.36 g/100 g d.m.) and dietary fiber (11.23–13.01 g/100 g d.m.) due to enzymatic modifications occurring during sprouting. Substantial reductions were observed in phytic acid (46%), tannins (63%), and trypsin inhibitors (54%), contributing to improved mineral content. Iron content increased from 9.53 to 14.49 mg/100 g, attributed to phytate degradation and concentration effects from dry matter loss. Functional attributes including seed imbibition, swelling power, and antioxidant activity improved, with antioxidant capacity rising from 63.22% to 85.81%. Overall, germination is an effective, low‐cost bioprocessing strategy to enhance nutritional quality and techno‐functional properties of barnyard millet for food applications.

## Graphical abstract

Germination is a simple and effective strategy to improve the nutritional and functional quality of millets. In this study, barnyard millet was germinated for 24, 48, and 72 h to evaluate its impact on nutrient composition, antioxidant properties, antinutritional factors, and functional characteristics. A significant reduction in phytic acid, tannin, and trypsin inhibitor was observed with increasing germination time. Concurrently, protein, fiber, total phenolic and flavonoid contents, antioxidant activity, and essential minerals (iron, calcium, and zinc) showed marked improvement. These findings highlight the potential of controlled germination to enhance the nutritional value and functional performance of barnyard millet for application in health‐oriented food products.

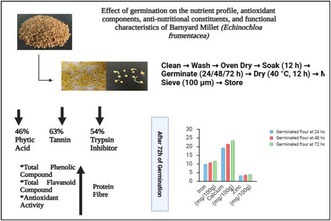

## 1 Introduction

Millets have served as a staple dietary constituent for human populations over thousands of years, particularly in arid and semiarid ecosystems, owing to their exceptional adaptability to adverse agroclimatic conditions and low resource requirements. Millets, belonging to the Poaceae family, are small‐seeded cereal crops traditionally cultivated for subsistence. Beyond their nutritional and ecological significance, millets constitute a critical component of agricultural systems in many developing countries, where they contribute substantially to food security and rural economies (Chandrasekara et al. 2012). Millets are often referred to as ‘famine reserves’ due to their exceptional storability, with grains remaining viable for consumption for up to 2 years or longer. In terms of global production, millets are ranked as the sixth most important cereal crop after rice, wheat, maize, sorghum, and barley (Ashoka et al. 2020). Among the diverse millet species, Barnyard millet ( *Echinochloa frumentacea* ) is an ancient cereal crop extensively cultivated across Asia, particularly in China, India, Korea, and Japan (Sood et al. 2014). Owing to its broad adaptability, the species thrives under diverse agroclimatic conditions and exhibits remarkable tolerance to drought and nutrient‐deficient soils, underscoring its potential as a climate‐resilient crop. Barnyard millet performs optimally in well‐drained sandy loam to loamy soils of moderate fertility and is characterized by low input requirements. Its short life cycle, with maturation occurring within 60–75 days, renders it highly suitable for short‐duration cultivation and crop diversification strategies (Roopashree Ugare et al. 2014). It is the fourth most widely cultivated minor millet, contributing significantly to food security among underprivileged populations worldwide (Renganathan et al. 2020). At the global level, India is the leading producer, with the largest cultivation area (~0.146 million hectares) and production volume (~0.147 million tonnes), achieving an average yield of 1034 kg/ha over the past 3 years (Iwakami et al. 2014). Barnyard millet contains approximately 68.8% carbohydrates, 10.1% protein, and 6.7% dietary fiber, and is a notable source of essential micronutrients, particularly zinc (3 mg/100 g) and iron (5 mg/100 g) (Kaur and Sharma 2020). Despite its rich nutrient profile, barnyard millet remains underutilized compared to staple cereals such as wheat and rice, primarily due to the presence of antinutritional factors including phytic acid, tannins, and oxalates, which limit the bioavailability of key micronutrients.

The presence of antinutritional compounds, including tannins, phytates, phenols, and enzyme inhibitors, reduces nutrient digestibility and mineral absorption, thereby constraining the nutritional potential of barnyard millet (Samtiya et al. 2020). The elevated tannin content of barnyard millet (64 mg/100 g), in conjunction with phytates, markedly reduces mineral bioavailability by forming insoluble complexes with divalent cations such as Iron (Fe), Calcium (Ca), and Magnesium (Mg). These interactions hinder intestinal absorption and consequently restrict the physiological utilization of these essential micronutrients (Onwurafor et al. 2020). Several preprocessing methods, including soaking, germination, and fermentation, have been recognized for their effectiveness in reducing antinutritional factors (ANFs) present in millets (Chethan Kumar et al. 2022). Germination is particularly effective in reducing antinutritional factors, thereby enhancing nutrient digestibility and bioavailability (Zakari 2015). During this process, enzymatic activity is triggered, initiating biochemical transformations under mild temperature conditions that support seedling development. Such modifications enhance the release of essential amino acids and minerals, improve protein and carbohydrate digestibility, increase antioxidant activity, and collectively contribute to an improved nutritional profile of barnyard millet (Azeez et al. 2022). Consequently, germination not only improves the nutritional quality of grains but also alters their physical and sensory characteristics, making them more suitable for addressing malnutrition and supporting general health (Chinma et al. 2015).

Research on the effects of germination has been extensively reported in cereals and certain minor millets; however, systematic investigations on barnyard millet remain scarce and are often restricted to basic compositional assessments. Integrated studies that simultaneously address nutrient dynamics, antioxidant constituents, antinutritional factors, and functional characteristics are particularly limited, despite barnyard millet being a nutrient‐dense, gluten‐free, and low‐glycemic millet with immense potential for addressing lifestyle‐related disorders such as diabetes and obesity. In contrast to earlier works that largely emphasize isolated parameters, this study adopts a comprehensive approach to elucidate the biochemical and functional transformations induced by germination, thereby bridging the gap between nutritional enhancement and practical applicability in food product development. With the escalating global demand for nutrient‐dense, sustainable, and climate‐resilient grains, there is an urgent need for cost‐effective processing strategies that not only improve nutritional quality but also enhance functional attributes. Germination, as a simple, eco‐friendly, and low‐cost bioprocessing technique, holds promise in this regard by reducing antinutritional constituents while simultaneously improving nutrient bioavailability and techno‐functional traits. This study systematically evaluates the influence of varying germination durations on the nutritional composition, antioxidant potential, antinutritional profile, and functional properties of barnyard millet. The findings are expected to provide novel insights into optimizing germination as a natural biofortification strategy and to support the wider utilization of germinated barnyard millet as a sustainable, health‐promoting alternative to conventional cereals within the food industry.

Therefore, this study aimed to comprehensively investigate the effect of varying germination durations (24, 48, and 72 h) on barnyard millet by examining changes in its physicochemical characteristics, functional properties, proximate composition, bioactive constituents, antioxidant activity, antinutritional factors, and mineral content. The study further sought to elucidate the structural modifications induced by germination using FTIR‐ATR analysis, thereby providing an integrated understanding of how germination enhances the nutritional quality and functional suitability of barnyard millet for food applications.

## 2 Material and Methods

### 2.1 Materials

Barnyard millet ( *Echinochloa frumentacea* ) was procured from the local market of Greater Noida, Uttar Pradesh, India in August 2024. All reagents used for this study were of analytical grade.

### 2.2 Sample Preparation

**FIGURE 1:**
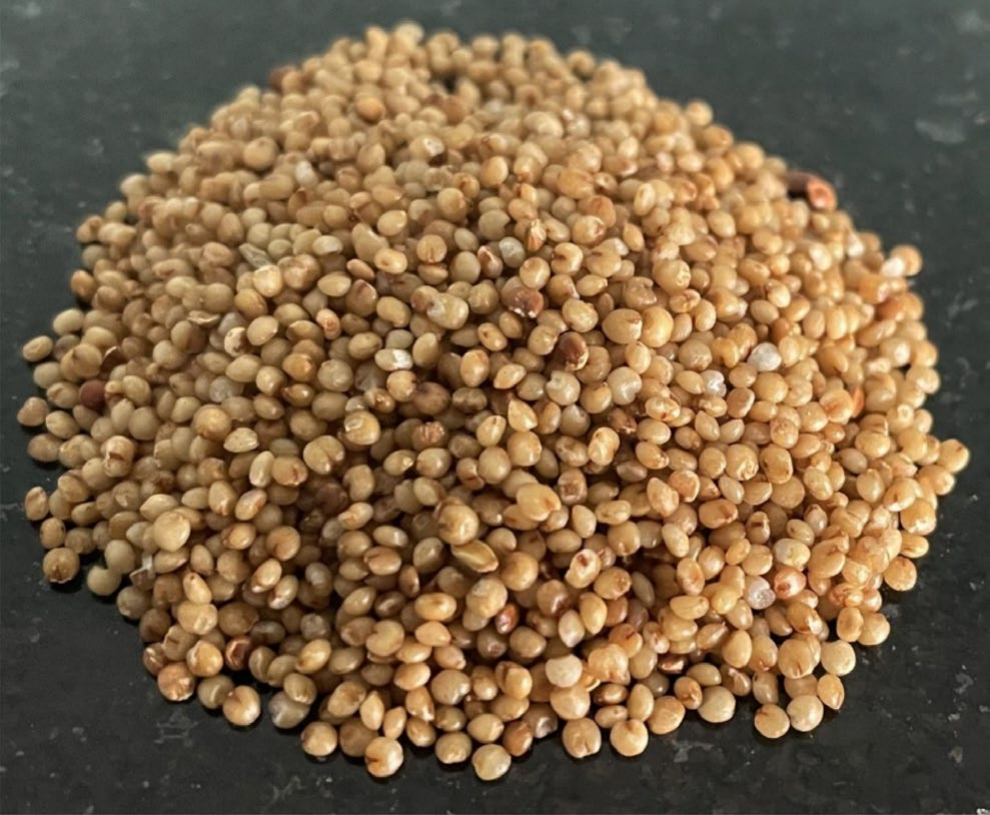
Raw barnyard millet grains.

**FIGURE 2:**
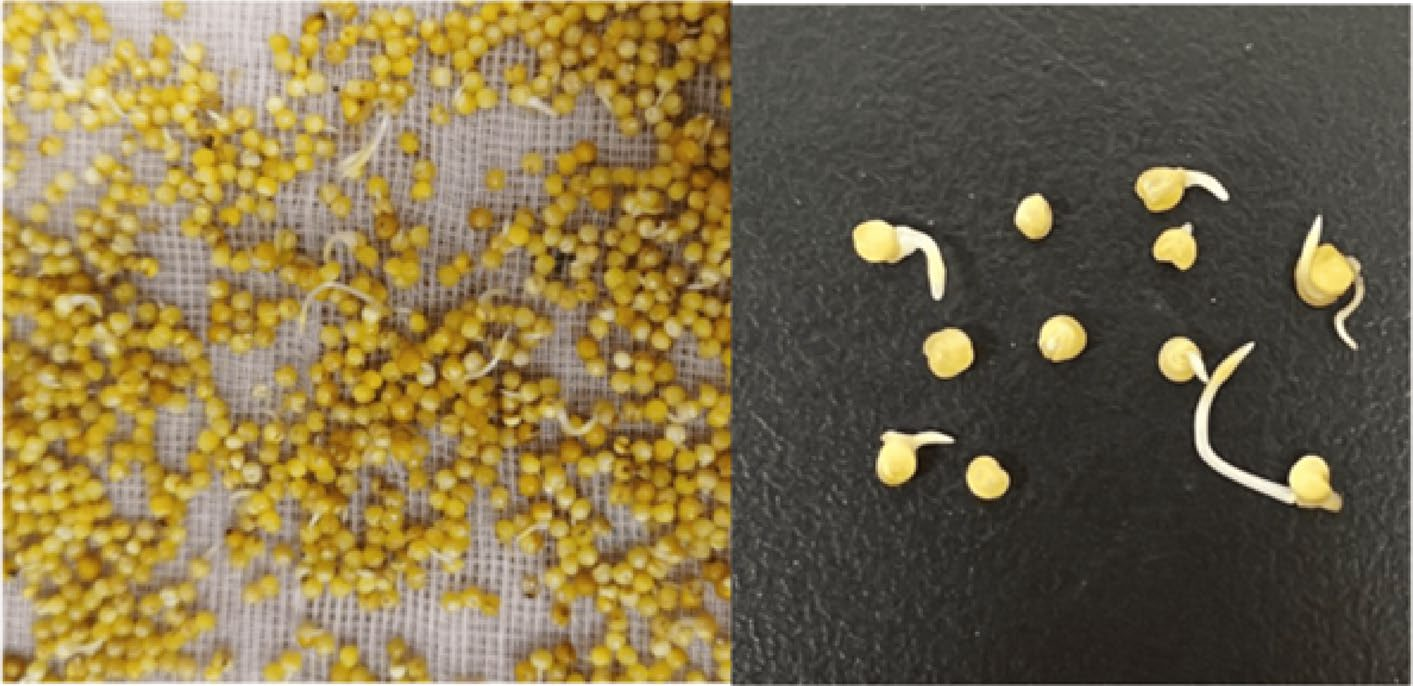
Germinated barnyard millet grains (72 h).

Raw barnyard millet grains were cleaned and washed, followed by oven drying at 40°C to remove surface moisture and standardize the samples. After drying, the moisture content was 10.26%. The dried grains were then used for all subsequent germination and analytical procedures. For germination, cleaned Barnyard millet was steeped in distilled water at 28°C ± 2°C for 12 h. After soaking, the grains were drained and arranged in a thin layer on moist blotting paper placed in perforated stainless‐steel trays, each covered with an identical tray. The trays were maintained at 28°C ± 1°C and 90% ± 1% relative humidity, and germination was carried out for three separate durations: 24, 48, and 72 h. At the end of each germination period, the grains were dried in a tray dryer at 40°C for 12 h, milled, and sieved through a 100 μm mesh to obtain germinated barnyard millet flour (GBMF). The flour was stored in sealed polypropylene bags placed in airtight containers at −4°C until further analysis. All compositional and mineral values are expressed on a dry matter (d.m.) basis unless otherwise stated (Figures 1 and 2).

### 2.3 Experimental Design

A completely randomized design was adopted with four germination treatments: 0 h (control), 24, 48, and 72 h. Each treatment consisted of an independent batch of 100 g of barnyard millet grains placed in a perforated stainless‐steel tray, and three independent batches were processed per treatment (biological replicates). All analytical measurements were carried out in technical triplicates.

For germination, grains were steeped in distilled water (3:1 water‐to‐grain ratio) at 28°C ± 2°C for 12 h. After draining, the grains were spread in a thin layer on moist blotting paper in perforated stainless‐steel trays and covered with identical trays to maintain moisture. The trays were kept under controlled laboratory conditions (28°C ± 1°C; ~90% relative humidity), and moisture was maintained by sprinkling distilled water as required. Trays were repositioned daily to minimize positional bias. Germination percentage and shoot length were assessed using 100 randomly selected seeds per batch, and dual counting was used to minimize observer bias. Seeds were considered germinated when the radicle length reached ≥ 2 mm. Any batch showing fungal growth or abnormal odor was discarded according to standard exclusion criteria. The sample size (*n* = 3 biological replicates) followed standard practice in cereal germination studies, ensuring adequate biological variation for statistical analysis. Processing steps—including drying in a tray dryer at 40°C for 12 h, milling, and sieving through a 100 μm mesh—were performed identically for all groups. The final moisture content after drying was 10.26%, ensuring uniform dry matter conditions prior to analysis.

### 2.4 Physicochemical Properties

#### 2.4.1 Assessment of Germination Loss, Shoot Length, and Germination Percentage

Grain loss during germination was evaluated following the methodology described by Yenasew and Urga (2023). The initial weight of the grains prior to germination (A) and the final weight after germination (B) were recorded, and the percentage loss was calculated using equation (1). Shoot length was recorded from randomly selected germinated barnyard millet seeds at 24, 48, and 72 h intervals. The average shoot length was determined across replicates. Germination percentage was calculated manually by counting the total number of seeds (*B*) and the number of seeds that successfully germinated (*A*) and applying the standard germination formula.

The mean shoot length was calculated across biological replicates. Germination percentage was determined manually by counting the total number of seeds (*B*) and the number of germinated seeds (*A*).

$$\begin{array}{c} \text{Total germination loss}\;\left(\%\right)-A-\frac{B}{B}\times 100\\ \text{Total germination percentage}-A/B\times 100 \end{array}$$

### 2.5 Determination of Functional Properties

Bulk density of the barnyard millet flour sample was carried out by following the method of (Yenasew and Urga 2023). The Water Absorption Capacity (WAC), Oil Absorption Capacity (OAC), Emulsion capacity (EC) and Foaming Capacity (FC) were determined following the procedure outlined by Azeez et al. (2022) with minor modification.

### 2.6 Estimation of Proximate Analysis

The nutritional composition of both germinated barnyard millet flours (GBMF) and Barnyard millet flour (BMF) was analyzed using standard analytical techniques. These included oven drying for moisture content, incinerating for ash determination, the Kjeldahl method for protein estimation, Soxhlet extraction for fat content, and crude fiber analysis, following the procedures outlined by AOAC (2010) (Horwitz and AOAC 2010).

### 2.7 Determination of Total Phenolic Content

Total phenolic content in both BMF and GBMF was estimated as per the method described by Chethan Kumar et al. (2022). A volume of 0.2 mL of Folin–Ciocalteu reagent was added to 0.5 mL of the sample supernatant in a test tube, followed immediately by the addition of 3.3 mL of distilled water and 1 mL of saturated sodium carbonate solution. The mixture was incubated for 30 min in the dark at room temperature to allow color development. The absorbance was then recorded at 700 nm using a spectrophotometer. Total phenolic content was quantified using a standard calibration curve prepared with gallic acid, and the results were expressed as milligrams of gallic acid equivalents (GAE) per 100 g of sample.

### 2.8 Determination of Total Flavonoid Content

The total flavonoid content of sample was determined using the method described by Azeez et al. (2022) with slight modification. Briefly, 0.3 mL of 5% Sodium Nitrate (NaNO)_2_ was added to 1 mL of extract which was transferred to a test tube. Following a 5 min incubation period, 0.3 mL of 10% Aluminum chloride (AlCl_3_) solution was added to the reaction mixture. After an additional 6 min, 2 mL of 1 M sodium hydroxide (NaOH) was introduced, and the final volume was adjusted to 10 mL using distilled water. The mixture was vortexed thoroughly to ensure complete mixing. Absorbance was measured immediately at 510 nm using a spectrophotometer. A calibration curve was prepared using a standard solution of gallic acid.

### 2.9 Determination of Antioxidant Activity

The antioxidant activity of BMF and GBMF extracts was quantified using the 2,2‐diphenyl‐1‐picrylhydrazyl (DPPH) radical scavenging assay following the method of Sharma et al. (2019). An aliquot of the extract was combined with a 1 M methanolic solution of 2,2‐diphenyl‐1‐picrylhydrazyl (DPPH) and incubated in the dark at room temperature for 30 min to ensure reaction stabilization. Subsequently, the absorbance was recorded at 517 nm using a spectrophotometer. The antioxidant activity was calculated as the percentage of DPPH radical scavenging in comparison to the control, using the following formula:

$$\text{Radical Scavenging Activity}-\left(A-B\right)/A\times 100$$

where *A* is the absorbance of the DPPH solution and *B* is the absorbance of the DPPH solution with the extract.

### 2.10 Determination of Antinutritional Factors

Tannin content was estimated by extracting 1 g of both the flours with 10 mL of acidified methanol (prepared by adding 1 mL of concentrated HCl to 100 mL of methanol). The mixture was incubated at 30°C for 15 h and then centrifuged for 15 min to obtain the supernatant. Simultaneously, the vanillin‐HCl reagent was prepared by combining equal volumes of vanillin solution (4 g vanillin in 100 mL methanol) and HCl‐methanol solution (8 mL concentrated HCl in 100 mL methanol). For the assay, 1 mL of the extract was mixed with 5 mL of vanillin‐HCl reagent and incubated in the dark for 20 min. Absorbance was measured at 500 nm using a UV–Visible spectrophotometer (Model AU 2701, Systronics, India). Tannin content was quantified using a standard calibration curve of tannic acid and expressed as milligrams of tannic acid equivalent (TAE) per 100 g of dry matter (d.m.) (Bhinder et al. 2019).

Total phytic content of the sample was determined using the method of Yadav et al. (2021). Phytic acid content was determined by extracting 0.1 g of the sample with 10 mL of 0.2 N hydrochloric acid in a centrifuge tube. The mixture was subjected to shaking at room temperature for 1 h to facilitate extraction, followed by centrifugation at 5000 rpm for 15 min. The resulting supernatant was collected for further analysis. For the colorimetric assay, 0.5 mL of the extract was reacted with 1 mL of ammonium ferric sulfate reagent, prepared by dissolving 100 mg of ammonium ferric sulfate in 50 mL of 0.2 N HCl and diluting the solution to 500 mL with distilled water. The mixture was heated in a boiling water bath for 30 min and subsequently cooled to 25°C. After cooling, 2 mL of bipyridine reagent composed of 1 g of bipyridine dissolved in 1 mL of thioglycolic acid and diluted to 100 mL with distilled water was added. The reaction mixture was allowed to stand for 2 min, and the absorbance was measured at 519 nm using a UV–Visible spectrophotometer (Model AU 2701, Systronics, India), with distilled water used as the blank. Quantification was performed using a standard curve prepared from a phytic acid sodium salt hydrate solution (130 mg in 100 mL of 0.2 N HCl), and results were expressed as milligrams of phytic acid per 100 g of d.m.

Trypsin inhibitor activity was assessed by extracting 1 g of flour sample with 0.01 M phosphate buffer (pH 7.5). The mixture was stirred continuously at room temperature for 1 h to facilitate extraction. Following centrifugation at 2000 × g for 30 min, the supernatant containing trypsin inhibitors was collected for analysis. For the assay, 50 μL of the extract, 50 μL of bovine trypsin solution, and 100 μL of 0.01 M Tris–HCl buffer (pH 7.5) were mixed and incubated at 37°C for 10 min. Subsequently, 100 μL of N‐α‐benzoyl‐DL‐arginine‐p‐nitroanilide (BAPNA) was added as the substrate, and the mixture was incubated for another 10 min at 37°C. The enzymatic reaction was terminated by adding 200 μL of 30% acetic acid. Absorbance was measured at 410 nm using a UV–Visible spectrophotometer to determine trypsin inhibitor activity (Sharma and Sahni 2021).

### 2.11 Minerals Estimation

Using a 3:1 ratio of HNO_3_ to HClO_4_, a mass of 1 g of barnyard millet flour sample was subjected to microwave‐assisted digestion. The digested sample was diluted with 50 mL of deionized water and was filtered using filter paper (Whatman no. 3). Mineral content was determined using Inductively Coupled Plasma–Mass Spectrometry (ICP–MS) with an X‐Series 2 instrument (Thermo Fisher Scientific, USA), following appropriate standard operating procedures for sample preparation and analysis. The expression for elements was mg/g for Fe, Ca, and Zn (Hymavathi et al. 2020).

### 2.12 Fourier Transform Infrared—Attenuated Total Reflection (FTIR‐ATR)

Fourier Transform Infrared Spectroscopy with Attenuated Total Reflectance (FTIR‐ATR) was employed to analyze both germinated and non‐germinated millet flour samples using a Bruker Vertex 70v spectrometer (USA). Spectral data were recorded over the range of 400 to 4000 cm^−1^ (Kulla et al. 2021).

### 2.13 Statistical Analysis

All experiments were conducted in triplicate, and the results were expressed as mean ± standard deviation. Statistical analysis was performed using one‐way ANOVA to assess differences among treatments, followed by Tukey's HSD post hoc test for multiple comparisons (*p* < 0.05). Data analysis was carried out using IBM SPSS Statistics software (version 17.0; SPSS Inc., Chicago, IL, USA).

## 3 Result and Discussion

### 3.1 Effect of Germination on Physiochemical Properties of Barnyard Millet Flour

The effects of germination on shoot length, germination percentage, and germination loss in barnyard millet are summarized in Table 1. Statistically significant differences (*p* < 0.05) were observed across all parameters with increasing germination duration.

**TABLE 1:** Effect of germination time on shoot length, germination percentage, and germination loss in barnyard millet flour.

Parameters	Control	24 h‐Germinated	48 h‐Germinated	72 h‐Germinated
Shoot length (cm)	0.32 ± 0.28ᵈ	1.41 ± 0.13ᶜ	3.08 ± 0.54ᵇ	4.29 ± 0.19ᵃ
Germination percentage (%)	96.24 ± 0.87ᵈ	96.69 ± 0.11ᶜ	97.18 ± 0.65ᵇ	97.87 ± 0.42ᵃ
Germination loss (%)	0.28 ± 0.19ᵈ	7.32 ± 0.55ᶜ	11.98 ± 0.39ᵇ	17.25 ± 0.21ᵃ

*Note:* Values are expressed as mean ± standard deviation (*n* = 3). Different superscript letters in a row indicate statistically significant differences (*p* < 0.05) using one‐way ANOVA and Tukey's HSD test.

Shoot length increased significantly (*p* < 0.05) with germination time. The shortest shoots were found in the control group (0.32 ± 0.28 cm), while the maximum length was recorded at 72 h (4.29 ± 0.19 cm). Each interval showed a statistically significant difference, indicating that even short germination periods notably enhanced seedling elongation. This trend supports the activation of growth‐related enzymes and improved seedling vigor during sprouting (Zakari 2015). Although high germination percentages were observed in all treatments, a statistically significant (*p* < 0.05) increase was noted over time. Germination percentage was lowest in the control group (96.24% ± 0.87%) and peaked at 72 h (97.87% ± 0.42%). These results demonstrate that optimal hydration and enzymatic activity during germination positively influence seed viability and successful sprouting (Chethan Kumar et al. 2022). Germination loss rose significantly (*p* < 0.05) with time. The control showed minimal loss (0.28% ± 0.19%), while the 72 h germinated samples exhibited the highest reduction in dry matter (17.25% ± 0.21%). Each increase in duration resulted in a statistically significant rise in weight loss, which can be attributed to the mobilization of stored nutrients to support metabolic activity and seedling development (Chinma et al. 2015).

### 3.2 Effect of Germination on Functional Properties of Barnyard Millet Flour

The functional properties of the control and germinated barnyard millet flour are summarized in Table 2. Bulk density is the measure of heaviness of the flour particles. Flour obtained from native Barnyard millet showed the highest bulk density, that is, 0.49 g/mL. Bulk density of the Barnyard flour decreased as germination time increased from 24 to 72 h and temperature from 25°C to 28°C. The bulk density decreased from 0.49 ± 0.34 to 0.28 ± 0.33 g/mL, which could be attributed to the modification of carbohydrates during germination and due to a decrease in heaviness and dispersibility of flour samples (Chinma et al. 2015). There was a significant decrease (*p* < 0.05) in the bulk density of barnyard millet flour at 24, 48, and 72 h of germination compared with the non‐germinated control. This reduction in bulk density may be attributed to structural loosening and the breakdown of complex compounds during germination, which increases porosity and reduces overall packing density (Ocheme et al. 2015).

**TABLE 2:** Effect of germination time on functional properties of Barnyard millet flour.

Conditions	Bulk density (g/mL)	Water absorption capacity (g/g)	Oil absorption capacity (g/g)	Emulsion capacity (%)	Foaming capacity (%)
Control	0.49 ± 0.34ᵃ	1.34 ± 0.61ᵈ	1.42 ± 0.73ᵈ	4.65 ± 0.18ᵈ	36.21 ± 0.11ᵈ
24 h Germinated sample	0.41 ± 0.12ᵇ	1.49 ± 0.18ᶜ	1.49 ± 0.31ᶜ	5.43 ± 0.19ᶜ	38.31 ± 0.29ᶜ
48 h Germinated sample	0.34 ± 0.66ᶜ	1.52 ± 0.48ᵇ	1.55 ± 0.79ᵇ	5.98 ± 0.25ᵇ	42.71 ± 0.28ᵇ
72 h Germinated sample	0.28 ± 0.33ᵈ	1.64 ± 0.18ᵃ	1.61 ± 0.41ᵃ	6.76 ± 0.33ᵃ	44.12 ± 0.70ᵃ

*Note:* Values are expressed as mean ± standard deviation (*n* = 3). Different superscript letters in a row indicate statistically significant differences (*p* < 0.05) using one‐way ANOVA and Tukey's HSD test.

Water absorption capacity of barnyard millet flour increased significantly (*p* < 0.05) after 24, 48, and 72 h of germination compared with the non‐germinated. The water absorption capacity of raw barnyard millet was 1.34 ± 0.61 g/100 g, which increased to 1.49 ± 0.18 g/100 g after 24 h of germination, 1.52 ± 0.48 g/100 g after 48 h, and reached a maximum of 1.64 ± 0.18 g/100 g at 72 h. The increase in water absorption capacity during germination can be attributed to structural modifications of existing macromolecules such as proteins and starch. Enzymatic hydrolysis and protein unfolding, along with partial degradation of starch and cell wall components, expose additional hydrophilic groups that enhance the water‐binding capacity of the flour (Gnanwa et al. 2021). Water retaining capacity was determined by the water‐binding capabilities of dietary components (Yenasew and Urga 2023). The significant water absorption capacity of GBMF implies that the flour might be effective in bakery products where hydration is necessary to increase functional and sensory qualities (Wani et al. 2020). A similar observation in increasing water absorbing capacity was reported by Sharma et al. (2018).

Oil absorption capacity (OAC) of raw BMF was 1.42 ± 0.73 g/100 g; however, after 72 h of germination, it increased to 1.61 ± 0.41 g/g. Agrawal (2013) stated that germination‐induced increased oil absorption capacity may be due to the solubilization and dissociation of proteins leading to exposure of nonpolar constituents within the protein molecule. There was a significant increase (*p* < 0.05) in the oil absorption capacity of barnyard millet at 24, 48, and 72 h germination relative to non‐germinated barnyard millet flour. The mechanism of oil absorption involves capillary action within the food matrix, which facilitates the retention of absorbed oil (Singh and Sharma 2017).

These results were in line with the report of Onwurafor et al. (2020), Ocheme et al. (2015). The results were 1.26 to 1.56 g/100 g in mung bean, 1.08 to 1.27 g/100 g in pea after 72 h of germination and in wheat there was an increase from 1.29 to 1.37 g/100 g, 1.26 to 1.39 g/100 g in maize and 1.27 to 1.38 g/100 g in sorghum after 48 h of germination. They reported an increase in OAC after germination of mung bean, pea, and wheat, maize, and sorghum, respectively.

Emulsion capacity of raw BMF seeds was found to be 4.65% ± 0.18%, which was increased to 6.76% ± 0.33% after 72 h of germination. The highest emulsion capacity values were recorded at 72 h germination. Sharma et al. (2016), reported that the dissociation and fractional unfolding of polypeptide chains occur during germination, exposing the hydrophobic sites of Amino Acid (AAs) which form complexes with lipid droplets, creating higher emulsion capacity. Emulsion capacity of barnyard millet flour increased significantly (*p* < 0.05) after 24, 48, and 72 h of germination compared with the non‐germinated. This enhancement may be attributed to improved stabilization of oil droplets at the interface, a function of food macromolecules. Denatured proteins contribute to this effect by generating electrostatic repulsion on the droplet surface, thereby promoting emulsion stability (Balwinder Singh et al. 2017).

The foaming capacity of raw BMF was found to be 36.21% ± 0.11%, which was significantly increased to 44.12% ± 0.70% with increase in germination time (72 h). Foaming properties were lower in the non‐germinated barnyard millet flour and higher in the 72 h germinated flour. There was a significant increase (*p* < 0.05) in the foaming capacity of Barnyard millet at 24, 48, and 72 h germination relative to non‐germinated barnyard millet flour. Germination resulted in a progressive increase in value, with a 5.79% rise after 24 h, 17.95% after 48 h, and the maximum enhancement of 21.84% observed at 72 h. This enhancement may be attributed to increased protein solubility in GBMF. The observed rise in foaming capacity could further result from conformational modifications of protein molecules, consistent with earlier findings (Sharma et al. 2019).

The improvements in water absorption, oil absorption, foaming, and emulsion capacities observed during germination enhance the technological suitability of barnyard millet flour for real‐world applications. Higher water absorption and swelling make germinated flour particularly suitable for infant foods, porridges, and gluten‐free bakery products requiring better hydration and softness (Wani et al. 2020). Improved oil absorption and emulsifying properties strengthen its use in baked goods, snacks, and batter formulations, where fat retention and stable emulsions are essential (Ma et al. 2020). These enhancements collectively expand the potential of germinated barnyard millet in developing nutritious and functional food products.

### 3.3 Effect of Germination on Proximate Composition of Barnyard Millet Flour

The proximate composition of BMF and GBMF are shown in Table 3. Moisture content of barnyard millet was increased during the process of germination. This rise is primarily due to hydration during soaking, which allows water uptake into the grain matrix. Additionally, structural modifications occurring during germination such as increased porosity and changes in sorption behavior enhance the ability of the grains to retain moisture (Yenasew and Urga 2023). The result obtained in this study was in line with the results obtained by Onwurafor et al. (2020), where the moisture content of finger millet increased from 14.29% to 26.21% during malting. Ash content decreased from 3.57% ± 0.23% to 3.07% ± 0.07% after 72 h of germination. This reduction may be attributed to the removal of shoots, roots, and bran layers, as well as the metabolic utilization of seed minerals during sprouting (Kulla et al. 2021). Similar to our result, Kumar et al. (2021) reported a decrease in the ash content of finger millet flour after germination, which was 2.27% and 1.24% in non‐germinated and germinated finger millet, respectively.

**TABLE 3:** Effect of germination time on proximate composition of Barnyard millet flour.

Conditions	Moisture (%)	Ash (%)	Fiber (%)	Fat (%)	Protein (%)
Control	10.26 ± 0.07ᵈ	3.57 ± 0.23ᵃ	11.23 ± 0.86ᵈ	4.39 ± 0.28ᵃ	9.21 ± 0.29ᵈ
24 h Germinated sample	18.94 ± 0.08ᶜ	3.25 ± 0.04ᵇ	12.04 ± 0.06ᶜ	4.03 ± 0.06ᵇ	9.99 ± 0.11ᶜ
48 h Germinated sample	19.42 ± 0.53ᵇ	3.17 ± 0.11ᶜ	13.59 ± 0.07ᵃ	3.11 ± 0.07ᶜ	11.29 ± 0.87ᵇ
72 h Germinated sample	20.18 ± 0.05ᵃ	3.07 ± 0.07ᶜ	13.01 ± 0.32ᵇ	2.02 ± 0.06ᵈ	13.36 ± 0.16ᵃ

*Note:* Values are mean ± standard deviation (*n* = 3). Different superscript letters within a row indicate significant differences at *p* < 0.05 using one‐way ANOVA followed by Tukey's HSD test.

There was a significant increase (*p* < 0.05) in the total dietary fiber of barnyard millet at 24, 48, and 72 h germination relative to non‐germinated barnyard millet flour. The total dietary fiber content in germinated barnyard millet increased from 11.23% ± 0.86% to 13.01% ± 0.32% after 72 h of germination. Prolonged germination significantly increased fiber content, likely due to structural modifications in cell wall polysaccharides, including alterations in tissue integrity and disruption of protein–carbohydrate interactions. These changes promote extensive cell wall biosynthesis, leading to the generation of additional dietary fiber (Setia et al. 2019).

The fat content of GBMF (4.39% ± 0.28%) decreased progressively with germination, declining to 4.03% ± 0.06%, 3.11% ± 0.07%, and 2.02% ± 0.06% after 24, 48, and 72 h, respectively. The reduction in fat content of malted flour may enhance shelf life by lowering susceptibility to rancidity, likely due to enzymatic activity during germination (Sharma et al. 2016). Similar reductions in fat content have been reported in barnyard millet by Abioye et al. (2018); and in finger millet by Dsouza (2013).

Germination markedly enhanced the protein content of barnyard millet flour, with increases of 8.46%, 22.58%, and 45.05% observed after 24, 48, and 72 h, respectively. The increase in protein content of barnyard millet with prolonged germination may be attributed to enhanced protease activity, which hydrolyses storage proteins into amino acids and peptides (Nkhata et al. 2018). This increment in protein content may also be ascribed to losses in dry weight, particularly in the carbohydrates through respiration during germination. The loss in dry weight can be due to loss in sugars during respiration as a result of production of carbon dioxide and water, which escaped from the seeds (Sharma et al. 2016). Mutshinyani et al. (2020) reported a significant increase in the protein content of finger millet during germination, rising from 11.26% to 18.21% after 72 h. Similarly, Chinma et al. (2015) and Azeez et al. (2022) observed an increase in the amount of protein after 72 h of germination in Bambara ground nut and brown finger millet flour, that is, 9.34% to 14.87% and 8.30% to 15.18%, respectively.

### 3.4 Effect of Germination on Total Phenolic Content of Barnyard Millet Flour

The TPC of non‐germinated barnyard millet flour was 40.70 ± 0.56 mg GAE/100 g d.m, which increased significantly (*p* < 0.05) to 91.32 ± 0.22 mg GAE/100 g d.m following 72 h of germination, indicating more than a two‐fold enhancement in phenolic constituents. The increase in total phenolic content in GBMF may be attributed to the activation of enzymes involved in the biosynthesis of phenolic compounds (Salawu et al. 2014). Salawu et al. (2014) demonstrated that germination of rye grains at varying temperatures over 6 days increased methanol‐extractable phenolic compounds, attributable to hydrolytic enzyme activation, cell wall structural modifications, and de novo synthesis of bioactive metabolites. Pradeep and Guha (2011) observed an increased phenol content in germinated little millet compared to its native form (non‐germinated whole millet), that is, 89.32 and 39.12 mg GAE/100 g d.m., respectively, after 48 h of germination.

### 3.5 Effect of Germination on Total Flavonoid Content of Barnyard Millet Flour

The flavonoid content of the barnyard millet was found to be increased during the process of germination. Total flavonoid content in raw barnyard millet was 22.21 ± 0.07 mg QE/100 g d.m., which increased to 27.96 ± 0.11, 39.06 ± 0.04, and 45.42 ± 0.08 mg QE/100 g d.m. after 24, 48, and 72 h of germination, respectively Munekata et al. (2016) reported a 34% increase in flavonoid content in chestnuts after 72 h of germination, likely due to germination‐induced biochemical changes that promote the synthesis of secondary metabolites. Accordingly, the observed increases in total phenolic content (TPC), total flavonoid content (TFC), and antioxidant activity of barnyard millet flour after germination may be attributed to enhanced phenolic compound accumulation (Mahbub et al. 2021).

### 3.6 Effect of Germination on Antioxidant Activity of Barnyard Millet Flour

Prolonged germination positively influenced the antioxidant activity of barnyard millet, which increased progressively with germination duration. The antioxidant activity of raw barnyard millet (63.22% ± 0.21%) increased significantly to 71.84% ± 0.32%, 77.39% ± 0.76%, and 85.81% ± 0.67% after 24, 48, and 72 h of germination, respectively. Germination significantly enhanced the radical scavenging activity of millet flours, likely due to the breakdown of high‐molecular‐weight polymers during sprouting, resulting in the formation of bioactive compounds (Singh et al. 2017). The observed enhancement in antioxidant activity during millet germination in this study may be attributed to the increased accumulation of phenolic compounds (PC) and flavonoid content (FC), which tend to rise progressively until reaching a saturation point. These findings highlight the critical role of optimizing germination duration to maximize antioxidant capacity, an important consideration for the development of millet‐based functional foods with enhanced health‐promoting properties (Table 4).

**TABLE 4:** Effect of germination time on bioactive compounds of barnyard millet flour.

Sample	TPC (mg GAE/100 g d.m.)	TFC (mg QE/100 g d.m.)	AoA (% DPPH)
Control	40.70 ± 0.56ᵈ	22.21 ± 0.07ᵈ	63.22 ± 0.21ᵈ
24 h Germinated	60.05 ± 0.04ᶜ	27.96 ± 0.11ᶜ	71.84 ± 0.32ᶜ
48 h Germinated	73.27 ± 0.27ᵇ	39.06 ± 0.04ᵇ	77.39 ± 0.76ᵇ
72 h Germinated	91.32 ± 0.22ᵃ	45.42 ± 0.08ᵃ	85.81 ± 0.67ᵃ

*Note:* Values are mean ± standard deviation (*n* = 3). Different superscript letters within a row indicate significant differences at *p* < 0.05 using one‐way ANOVA followed by Tukey's HSD test.

### 3.7 Effect of Germination on Antinutritional Factor of Barnyard Millet Flour

**FIGURE 3:**
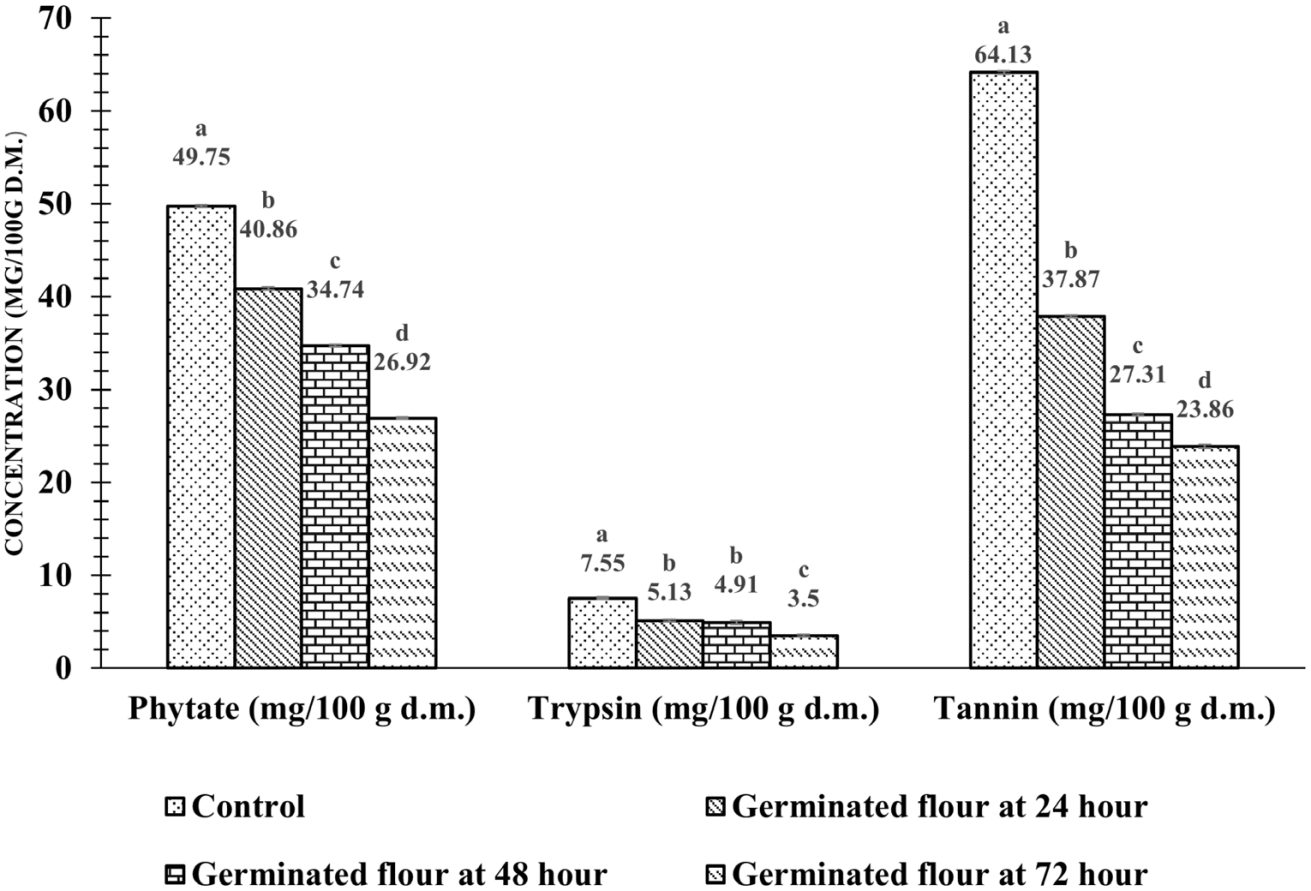
Effect of germination duration on antinutritional factors in barnyard millet flour. Values are presented as mean ± SD (*n* = 3). Bars with different letters within each parameter indicate significant differences at *p* < 0.05, as determined by Tukey's HSD post hoc test.

The tannin, phytate contents and trypsin inhibitor activity in barnyard millet flour was found to be decreased during the germination. The antinutritional factors of raw and germinated barnyard millet flour ar shown in Figure 3. After 72 h of germination, tannin content decreased from 64.13 ± 0.18 to 23.86 ± 0.24 mg/100 g, representing a reduction of approximately 63%. Phytate content decreased from 49.75 ± 0.11 to 26.92 ± 0.44 mg/100 g (≈46% reduction), while trypsin inhibitor activity decreased from 7.55 ± 0.04 to 3.50 ± 0.19 mg/100 g (≈54% reduction).

In this study, a reduction in antinutritional factors was observed with increasing germination time, which may be attributed to the enhanced hydrolytic activity of the enzyme phytase during the time of germination (Sinha and Kawatra 2003). During germination, phytate phosphorus undergoes hydrolysis to inositol monophosphate due to increased phytase activity, leading to a reduction in phytic acid content. A significant decline in tannin levels was also recorded, which may be attributed to the hydrophobic interactions formed between tannins and seed proteins or enzymes. Additionally, the reduction in tannin content could result from leaching into the soaking medium or binding of polyphenols with other organic constituents such as carbohydrates or proteins (Ohanenye et al. 2020). The observed decrease in trypsin inhibitor activity during germination may be attributed to its utilization as an energy source and degradation by peptic and pancreatic hydrolytic enzymes (Bhagwan et al. 2016). This reduction is advantageous, as it enhances the protein digestibility of germinated millets, thereby increasing their overall nutritional value. The observed decline in trypsin inhibitor activity is consistent with earlier reports demonstrating that germination effectively reduces antinutritional factors in cereal grains (Majzoobi et al. 2023). Ma et al. (2020) demonstrated that 72 h of germination markedly reduced the levels of tannins (54.23–22.14 mg/100 g), phytic acid (58.23–18.16 mg/100 g), and trypsin inhibitor activity (9.25–2.52 mg/100 g) in soybean. Mutshinyani et al. (2020) likewise reported that 72 h of germination significantly decreased tannin (49.21–19.23 mg/100 g), phytic acid (64.13–31.21 mg/100 g), and trypsin inhibitor activity (8.17–1.23 mg/100 g) in finger millet.

### 3.8 Effect of Germination on Mineral Content of Barnyard Millet Flour

**FIGURE 4:**
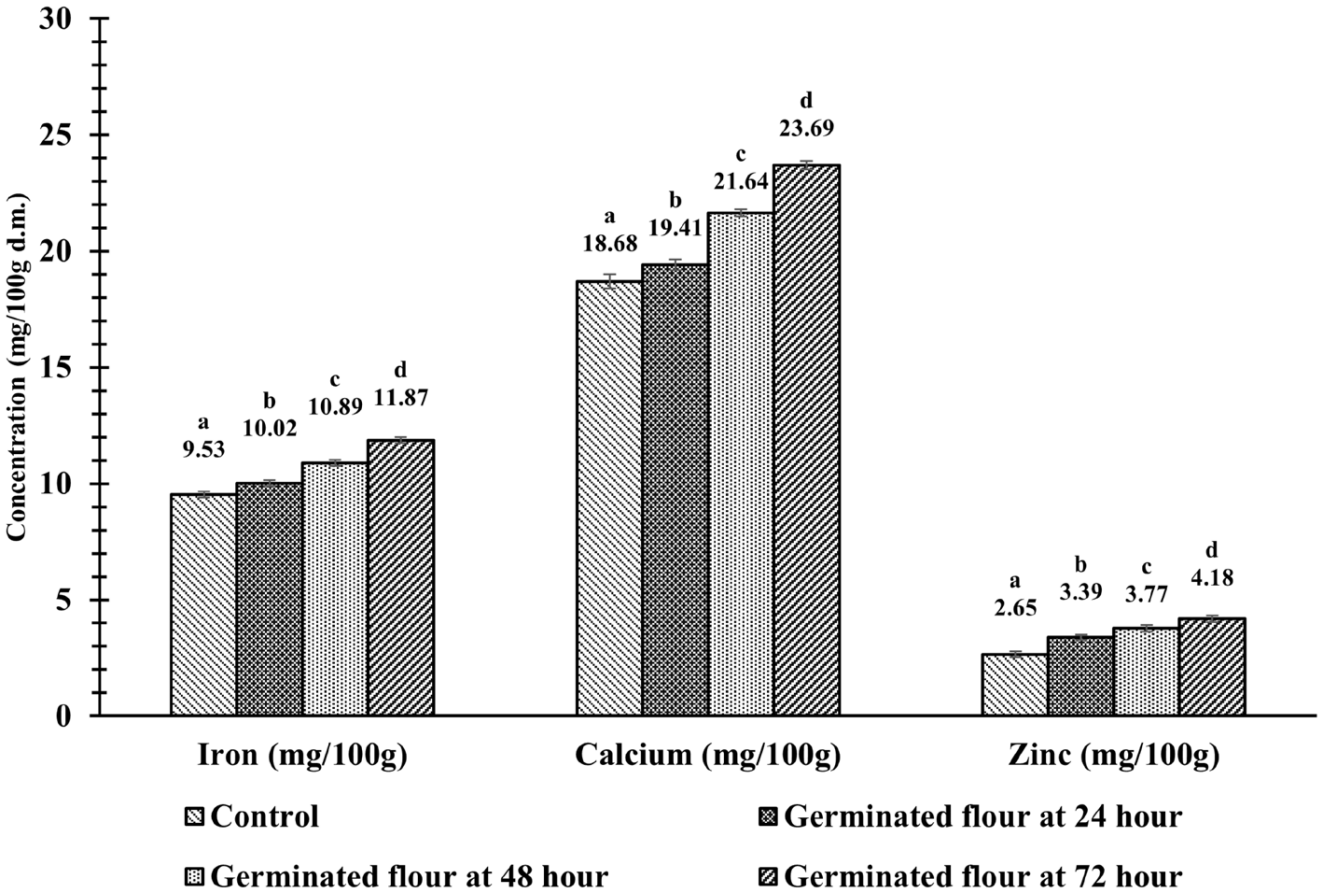
Effect of germination duration on mineral content in barnyard millet flour. Values are presented as mean ± SD (*n* = 3). Bars with different letters within each parameter indicate significant differences at *p* < 0.05, as determined by Tukey's HSD post hoc test.

The impact of germination on the mineral content on barnyard millet is depicted in Figure 4. The germination process is assumed to increase the mineral content due to an increase in phytase enzyme activity. The phytase enzyme hydrolyses the bond between the phytic acid and trapped minerals; hence, the minerals become free, increasing their availability (Narayanan and Lakshmanaswamy 2017). Germination enhanced the mineral profile of barnyard millet flour, with notable increases in Fe, Ca, and Zn contents. Specifically, Fe content increased from 9.54 ± 0.18 to 11.87 ± 0.32 mg/100 g after 72 h of germination.

The Fe content of barnyard millet increased with prolonged germination, primarily attributed to enhanced phytase activity. This enzyme hydrolyses protein–mineral complexes, thereby releasing bound minerals and improving their bioavailability (Azeez et al. 2022). Calcium content of Barnyard millet flour was found to be increased with the increased germination time. The Ca content of raw barnyard millet flour (18.68 ± 0.09 mg/100 g) increased to 23.69 ± 0.22 mg/100 g after 72 h of germination. This trend aligns with the findings of (Suma and Urooj 2014), who reported a similar enhancement of Ca in pearl millet, after 72 h of germination. The increase observed in our study may be attributed to germination‐induced biochemical changes that enhance mineral availability, rather than dry matter loss, which is typically associated with increases in minerals such as Zn.

Similarly, the Zn content also increased after germination; in raw flour it was 2.65 ± 0.28 mg/100 g and after 72 h germination it increased to 4.18 ± 0.21 mg/100 g. The increase in Ca in germinated flour might be due to the loss of organic dry matter from the grains during germination and this increases the percentage of Ca in the flour (Afify et al. 2012). The reduction in oxalic acid during germination corresponded with an increase in Ca content in millet, as oxalic acid is known to chelate calcium and hinder its absorption (Kulla et al. 2021). Similar trends have been reported by Jan et al. (2017), Chethan Kumar et al. (2022), and Suma and Urooj (2014), who observed increased mineral contents in Chenopodium, sorghum, maize, and pearl millet. These authors further reported that germination enhanced Fe content: from 5.76 to 8.31 mg/100 g in Chenopodium (72 h), 3.47 to 7.21 mg/100 g in sorghum (72 h), 2.87 to 4.76 mg/100 g in maize (48 h), and 7.32 to 10.34 mg/100 g in pearl millet (48 h).

A significant inverse relationship was observed between antinutritional factors tannins, phytic acid, and trypsin inhibitors and the Fe and Ca content of barnyard millet during germination. The reduction of these antinutrients, which impede mineral absorption, corresponded with enhanced mineral bioavailability (Afify et al. 2012). Phytic acid, a major chelator of divalent cations, forms insoluble complexes with Fe and Ca, limiting their intestinal uptake. However, the activation of endogenous phytase during germination facilitates the hydrolysis of phytic acid into lower inositol phosphates, thereby reducing its chelating potential and enhancing mineral release (Gnanwa et al. 2021). The observed increases in Fe and Ca content in germinated barnyard millet may also result from the breakdown of complex antinutrient mineral matrices and the loss of dry matter, which effectively concentrates minerals on a per‐gram basis. These changes collectively enhance mineral bioavailability, highlighting germination as a cost‐effective and efficient strategy to reduce antinutritional factors while improving the nutritional quality of barnyard millet (Hymavathi et al. 2020).

The biochemical and nutritional enhancements observed during germination have important implications for real‐world food applications (Jan et al. 2017). The increases in protein, dietary fiber, mineral availability, and antioxidant constituents can contribute to improved nutritional quality and enhanced health benefits, such as better digestibility, reduced glycemic response, and improved micronutrient bioavailability particularly relevant for populations with iron‐ or calcium‐deficiency risks (Gnanwa et al. 2021). The improved functional properties, including higher water absorption, oil absorption, and emulsifying capacity, make germinated barnyard millet flour suitable for bakery products, porridges, weaning foods, extruded snacks, and gluten‐free formulations (Williams Erickson et al. 2018). To retain these benefits during processing and storage, strategies such as low temperature drying, minimal heat treatment, controlled moisture reduction, and storage under low humidity and oxygen conditions are recommended. Incorporating antioxidants, using whole‐grain formats, and avoiding excessive thermal processing may further help preserve phenolics, flavonoids, and functional attributes. These approaches ensure that the nutritional and functional improvements obtained through germination are effectively translated into stable, high‐quality food products (Ab Rahman et al. 2018).

### 3.9 FTIR‐ATR Analysis

**FIGURE 5:**
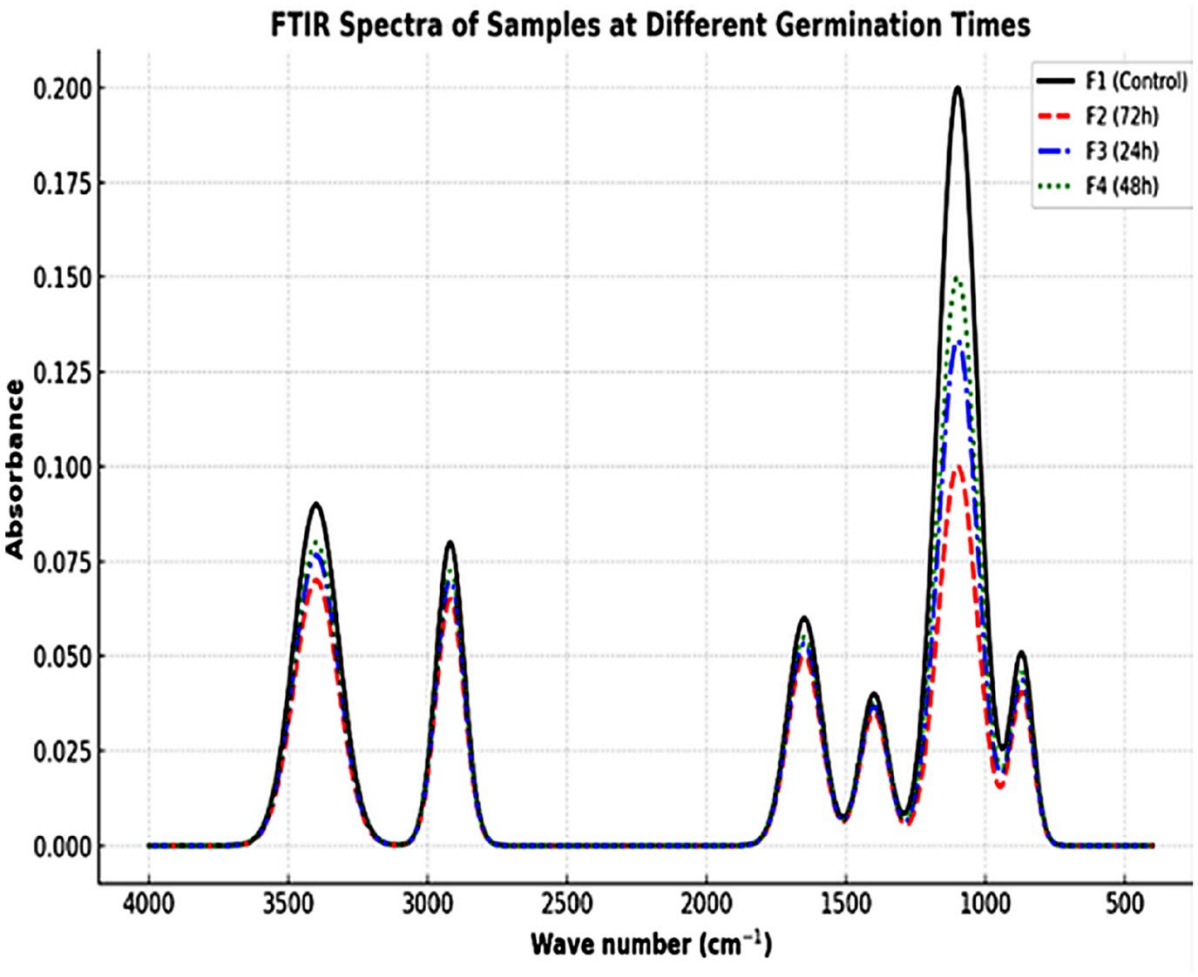
FTIR‐ATR spectra of raw and germinated barnyard millet flour.

The FTIR spectra of barnyard millet samples, including the control (F1) and germinated samples at 24 h (F3), 48 h (F4), and 72 h (F2), are presented in Figure 5. Germination induced significant spectral changes, reflecting alterations in the chemical composition and functional properties of barnyard millet samples (Setia et al. 2019). A broad peak observed around 3300–3400 cm^−1^ corresponds to –OH and –NH stretching vibrations, indicative of hydroxyl groups in phenolic compounds and proteins. The F2 (72 h) sample exhibited the highest spectral intensity in this region, indicating an increased concentration of bioactive compounds and enhanced protein solubility, likely resulting from intensified enzymatic activity and cell wall degradation during the extended germination period (Siddiqua et al. 2019). The peak near 2920 cm^−1^, corresponding to C–H stretching of lipids, exhibited reduced intensity in F2 relative to the control, suggesting the utilization of lipid reserves during germination, a typical metabolic adaptation in sprouting seeds (Chethan Kumar et al. 2022).

In the amide I region (~1650 cm^−1^), related to C = O stretching of proteins, F2 displayed a more pronounced absorption than other germinated samples. This reflects protein unfolding and enhanced proteolytic activity, suggesting improved digestibility and nutritional bioavailability (Beasley et al. 2019). The fingerprint region (1400–1000 cm^−1^), mainly involving C–O and C–N vibrations from carbohydrates and proteins, showed more refined and lower intensity peaks in F2. This reduction can be attributed to depolymerization of complex carbohydrates and increased release of soluble sugars, confirming efficient biochemical conversion (Narayanan and Lakshmanaswamy 2017). Notably, the absorbance in the region of 870–900 cm^−1^, often linked to β‐glucans and structural polysaccharides, significantly decreased in F2. This implies breakdown of structural cell wall components, further enhancing nutrient accessibility (Ohanenye et al. 2020). Among all formulations, F2 (72 h germinated sample) demonstrated the most favorable spectral shifts, indicating enhanced nutritional composition, improved protein structure, breakdown of antinutritional fibers, and release of bioactive components. This aligns with literature suggesting that extended germination maximizes biochemical conversions, making F2 the most nutritionally enriched and functionally superior sample in the study (Narayanan and Lakshmanaswamy 2017).

The improvements observed in barnyard millet during germination follow patterns commonly reported in other small millets such as finger millet, foxtail millet, and sorghum. However, the extent of reduction in antinutritional factors and the enhancement in functional and nutritional attributes were comparatively more pronounced in barnyard millet. This indicates that barnyard millet responds particularly well to germination, making it a promising grain for developing nutrient‐dense germinated food products. The findings of this study also contribute to the advancement of sustainable food systems. Germination is a low‐cost, low‐energy, and environmentally friendly processing technique that enhances the nutritional quality of underutilized grains such as barnyard millet, reducing dependence on resource‐intensive cereals. Promoting germinated millet‐based products can support climate‐resilient agriculture, as millets require minimal water, tolerate harsh climatic conditions, and grow well in marginal soils, thereby strengthening food security in vulnerable regions. From a socioeconomic perspective, value addition through germination can create new market opportunities for farmers and small‐scale food processors, contributing to rural livelihoods and local economies. Future interdisciplinary research integrating food science, agronomy, microbiology, and consumer sciences is needed to optimize germination parameters, evaluate product stability, assess sensory acceptance, and explore large‐scale production models. Life cycle assessments and techno‐economic evaluations will further help quantify environmental and economic impacts, guiding the development of sustainable, nutrient‐dense, millet‐based functional foods.

## 4 Conclusion

The findings of this study demonstrate that germination substantially enhances the nutritional profile, functional characteristics, and antioxidant capacity of barnyard millet. Soaking for 12 h followed by germination for 24–72 h was effective in significantly reducing antinutritional factors such as tannins and phytic acid, thereby improving the total content of essential minerals. Notably, extended germination for up to 72 h resulted in marked improvements in proximate composition, functional attributes, and antioxidant potential. These enhancements affirm the role of germination in augmenting the nutritional and functional quality of barnyard millet. Additionally, FTIR‐ATR analysis revealed distinct changes in functional group regions, suggesting chemical modifications associated with the germination process. Collectively, these results highlight the potential of germinated barnyard millet as a functional ingredient for the formulation of nutritionally enriched food products, including noodles, pasta, and weaning foods, thus contributing to the diversification and enhancement of millet‐based diets.

## Author Contributions

**Meena Kumari:** supervision, writing – review and editing. **Rachna Sehrawat:** supervision, writing – review and editing. **Aina Chaudhary:** conceptualization, writing – original draft. **Lokesh Kumar:** writing – review and editing.

## Ethics Statement

The authors have nothing to report.

## Conflicts of Interest

The authors declare no conflicts of interest.

## Data Availability

The data that support the findings of this study are available from the corresponding author upon reasonable request.
